# The Effect of Methylmalonic Acid Treatment on Human Neuronal Cell Coenzyme Q_10_ Status and Mitochondrial Function

**DOI:** 10.3390/ijms21239137

**Published:** 2020-11-30

**Authors:** Emma C. Proctor, Nadia Turton, Elle Jo Boan, Emily Bennett, Suzannah Philips, Robert A. Heaton, Iain P. Hargreaves

**Affiliations:** 1 Department of Biochemistry, University of Warwick, Coventry CV4 7AL, UK; Emma.Proctor@warwick.ac.uk; 2 School of Pharmacy and Biomolecular Sciences, Liverpool John Moores University, Liverpool L3 3AF, UK; N.M.Turton@2020.ljmu.ac.uk (N.T.); elleboan212@gmail.com (E.J.B.); Emily-Ben-97@hotmail.co.uk (E.B.); R.Heaton@2013.ljmu.ac.uk (R.A.H.); 3 Department of Clinical Biochemistry, The Royal Liverpool University Hospital, Royal Liverpool and Broadgreen NHS Trust, Prescot Street, Liverpool L7 8XP, UK; Suzannah.Phillips@liverpoolft.nhs.uk

**Keywords:** coenzyme Q_10_, mitochondrial respiratory chain, methylmalonic acidemia, mitochondrial dysfunction, mitochondrial membrane potential

## Abstract

Methylmalonic acidemia is an inborn metabolic disease of propionate catabolism, biochemically characterized by accumulation of methylmalonic acid (MMA) to millimolar concentrations in tissues and body fluids. However, MMA’s role in the pathophysiology of the disorder and its status as a “toxic intermediate” is unclear, despite evidence for its ability to compromise antioxidant defenses and induce mitochondrial dysfunction. Coenzyme Q_10_ (CoQ_10_) is a prominent electron carrier in the mitochondrial respiratory chain (MRC) and a lipid-soluble antioxidant which has been reported to be deficient in patient-derived fibroblasts and renal tissue from an animal model of the disease. However, at present, it is uncertain which factors are responsible for inducing this CoQ_10_ deficiency or the effect of this deficit in CoQ_10_ status on mitochondrial function. Therefore, in this study, we investigated the potential of MMA, the principal metabolite that accumulates in methylmalonic acidemia, to induce a cellular CoQ_10_ deficiency. In view of the severe neurological presentation of patients with this condition, human neuroblastoma SH-SY5Y cells were used as a neuronal cell model for this investigation. Following treatment with pathological concentrations of MMA (>0.5 mM), we found a significant (*p* = 0.0087) ~75% reduction in neuronal cell CoQ_10_ status together with a significant (*p* = 0.0099) decrease in MRC complex II–III activity at higher concentrations (>2 mM). The deficits in neuronal CoQ_10_ status and MRC complex II–III activity were associated with a loss of cell viability. However, no significant impairment of mitochondrial membrane potential (ΔΨm) was detectable. These findings indicate the potential of pathological concentrations of MMA to induce a neuronal cell CoQ_10_ deficiency with an associated loss of MRC complex II–III activity. However, in the absence of an impairment of ΔΨm, the contribution this potential deficit in cellular CoQ_10_ status makes towards the disease pathophysiology methylmalonic acidemia has yet to be fully elucidated.

## 1. Introduction

Methylmalonic acidemia is an organic acidemia which although clinically difficult to manage can show improvement as the result of treatment. This condition can result from either a deficiency in l-methylmalonyl-CoA mutase (MCM; MIM 251000) activity, a deficit in 5-deoxyadenosyl cobalamin (the active form of vitamin B12) synthesis or availability (MIM 251110), and in rare cases it can be caused by a deficiency in the activity of methylmalonyl-CoA epimerase (MCE; MEM 251120) [1]. Biochemically, methylmalonic acidemia is characterized by the accumulation of methylmalonic acid (MMA) in tissues and body fluids, such as blood plasma or serum and cerebrospinal fluid (CSF), although the majority of biochemical studies use urine to determine MMA levels in patients [2]. The two major disease phenotypes are isolated methylmalonic acidemia and combined methylmalonic acidemia with homocystinuria. The isolated variant of this condition can be caused by either a complete deficiency (enzymatic subtype mut^0^) or a partial deficiency (subtype mut^-^) in MCM activity, or can result from defects in the transport or synthesis of adenosylcobalamin (cblA and cblB). As a result of these deficiencies, the catabolic pathway of branched chain amino acids is impaired in methylmalonic acidemia [1,3].

Methylmalonic acidemia presents in infancy with episodes of metabolic acidosis, although this condition can also present in adulthood [1,3]. Damage to the central nervous system which can present as developmental delay and coma, together with chronic kidney disease are amongst the severe consequences of this disease, although this condition can also be fatal in some cases [1,3]. However, disease severity and individual symptoms can vary, ranging from a benign clinical presentation to a fatal neonatal syndrome [4]. A comprehensive overview of the clinical presentations of methylmalonic acidemia is outlined by Baumgartner et al. (2014) [4]. Despite recent management guidelines for the treatment of methylmalonic acidemia this disorder is associated with significant morbidity and mortality [4]. Athough some patients with methylmalonic acidemia may respond to cobalamin therapy, in severe cases of the disease the transplantation of affected organs such as the liver and kidney may be considered in order to overcome local enzyme deficiencies [5]. However, neurological deterioration post transplant is common, and patients remain susceptible to a “metabolic stroke”, with CSF MMA levels remaining significantly high post liver transplantation, despite reduction in serum levels [6,7]. The commonly used therapy for the treatment of patients with methylmalonic acidemia involves the dietary restriction of natural proteins to limit the intake of isoleucine, valine, methionine, and threonine together with the provision of a high energy diet, although this can result in amino acid deficiencies and has been associated with poor growth [8,9].

MMA has been previously reported to induce neuron apoptosis and oxidative stress (OS) in different cell culture systems [10,11,12]. Furthermore, the mitochondrial toxicity of MMA has been examined in both in vitro and in vivo systems [13]. MMA has also been reported to induce an increase in lipid and protein peroxidation as well as to decrease the activity of the antioxidant enzyme, superoxide dismutase in rat brain and kidney tissue, the pro-oxidant effect of MMA was found to be potentiated by the induction of renal failure [11,14]. The metabolites 2-methylcitrate (2-MCA) and malonate (MA) have also been reported to accumulate in methylmalonic acidemia together with MMA and have been implicated in neuronal death and MRC impairment, although their mechanism of actions have yet to be fully elucidated [3,15].

A number of studies have demonstrated evidence of both single and multiple mitochondrial respiratory chain (MRC) enzyme deficiencies in patients with methylmalonic acidemia [16,17,18]. An in vitro study using rat cerebra cortex reported that MRC complex II–III (NADH: cytochrome *c* reductase; EC: 1.6.5.3 + EC: 1.10.2.2) and complex II–III (succinate: cytochrome *c* reductase; EC: 1.3.5.1 + EC: 1.10.2.2) activities were significantly reduced following treatment with 1, 2.5 and 5 mM MMA [19]. Although the activity of MRC complex I (NADH:ubiquinone reductase; EC: 1.6.5.3) was also found to be decreased following MMA exposure, the activities of individual MRC II (succinate dehydrogenase: ubiquinone reductase; EC: 1.3.5.1) and complex III (ubiquinol: cytochrome *c* reductase; EC: 1.10.2.2) were not affected [19]. Therefore, in view of the dependence of MRC complex II–III activity upon endogenous coenzyme Q_10_ (CoQ_10_), the decrease in the activity of this enzyme following MMA exposure may have resulted from a deficit in CoQ_10_ status induced by this metabolite [20]. However, this possibility was not further investigated in the study by Brusque et al. (2002) [19].

CoQ_10_ serves as an electron carrier within the MRC as well as functioning as an important lipid-soluble antioxidant [21]. In its role as an electron carrier within the MRC, CoQ_10_ allows a continuous passage of electron in the chain enabling the process of oxidative phosphorylation to occur with the concomitant production of ATP [21]. The antioxidant function of CoQ_10_ is attributed to its fully reduced ubiquinol form. In addition to its electron carrier and antioxidant functions, CoQ_10_ has also been reported to have anti-inflammatory properties and be involvement in DNA replication and repair [22,23].

Haas et al. (2009) [24] revealed evidence of a deficit in fibroblast CoQ_10_ status in patients with methylmalonic acidemia who demonstrated no residual MCM activity [24]. A further study reported a decreased in the level of renal CoQ_10_ in a MUT-knockout mouse model of methylmalonic acidemia. However, the level of coenzyme Q_9_ (CoQ_9_) which is the predominant ubiquinone species in mouse [20] was comparable to control levels [25]. At present, only two studies have reported evidence of an association between a CoQ_10_ deficiency and methylmalonic acidemia, and the factors responsible for eliciting this deficit in cellular CoQ_10_ status have yet to be investigated. In view of the possibility that the CoQ_10_ deficiency associated methylmalonic acidemia may be caused by one of the metabolites that accumulate in this condition, a possible candidate to consider would be MMA since this is the principal compound that accumulates to mM concentrations in the body fluids and brain tissue of patients with methylmalonic acidemia during acute metabolic crisis [19,26]. Therefore, the aims of this study were to investigate the potential of MMA to induce a cellular CoQ_10_ deficiency as well as to provide further evidence supporting an association between this condition and a deficit in endogenous CoQ_10_ status and mitochondrial dysfunction. In view of the severe neurological presentation of patients with methylmalonic acidemia [13], human neuroblastoma SH-SY5Y cells were chosen as the appropriate cell line for this investigation.

## 2. Results

### 2.1. The Effect of MMA Treatment on Neuronal CoQ_10_ Status and MRC Complex II–III Activity

To assess the effect of MMA treatment on the neuronal MMA status, and to confirm that this metabolite was taken up by the SH-SY5Y cells, the intracellular concentration of MMA was analysed by liquid chromatography–tandem mass spectrometry (LC-MS) following 7 days of culture with exogenous MMA (2 and 5 mM). Intracellular concentrations of MMA of 3.01 ± 1.14 nmol/mg and 5.28 ± 0.25 nmol/mg (mean ± SD; *n* = 3) were determined following 7 days of incubation with 2 mM and 5 mM, respectively. These results indicated that, interestingly, control cells were found have an endogenous MMA concentration of 0.64 ± 0.16 nmol/mg (mean ± SD; *n* = 3).

**Figure 1:**
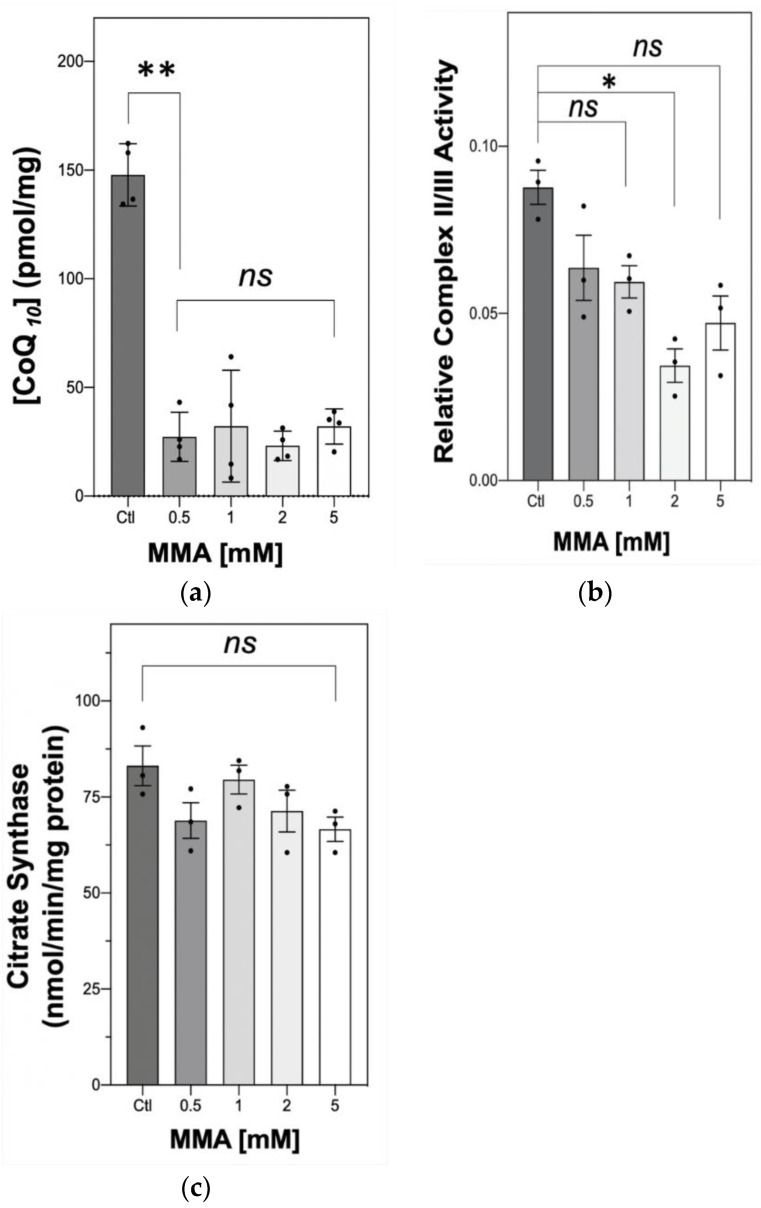
Effect of MMA treatment (0.5, 1, 2 and 5 mM) on neuronal cell CoQ_10_ status, the cellular CoQ10 concentration is expressed as pmol/mg of protein (**a**). Effect of MMA treatment (0.5, 1, 2 and 5 mM) on neuronal cell MRC complex II–III activity (**b**). Effect of MMA treatment (0.5, 1, 2 and 5 mM) on neuronal cell CS activity (**c**). Error bars represent the mean ± SEM of *n* = 4 observations (**a**) and *n* = 3 (**b**,**c**). Statistical analysis was carried out using the Kruskal–Wallis test with Dunn’s multiple comparisons. ** *p* = 0.0087, * *p* = 0.0099.

Following incubation with MMA, neuronal CoQ_10_ status was found to be significantly decreased (** *p* = 0.0087, Figure 1a) compared to control levels for all treatment groups (0.5, 1, 2 and 5 mM MMA). Interestingly, although 0.5 mM MMA induced an approximate 75% decrease in cellular CoQ_10_ status, the treatment of the SH-SY5Y cells with higher concentrations of MMA (1, 2, 5 mM; Figure 1a) did not result in a further deficit in cellular CoQ_10_ status which was decreased to a similar level following all treatments.

The effect of this deficit in neuronal CoQ_10_ status was then investigated on MRC activity by determining the activity of MRC complex II–III, a linked enzyme system whose activity is dependent upon endogenous CoQ_10_ status [20]. MMA treatment was found to decrease complex II–III activity, although this only reached significance at 2 mM (* *p* = 0.0099, Figure 1b). The effect of MMA treatment upon the activity of the mitochondrial marker enzyme, citrate synthase (CS, EC. 2.3.3.8) [27] was also undertaken to ensure that the diminution in neuronal CoQ_10_ status did not simply reflect a loss in mitochondrial enrichment since approximately 50% of cellular CoQ_10_ is located within this organelle [28]. No significant changes were found between the concentration range (ns, *p* = 0.081, Figure 1c).

### 2.2. The Effect MMA Treatment on SH-SY5Y Viability and Mitochondrial Membrane Potential

**Figure 2:**
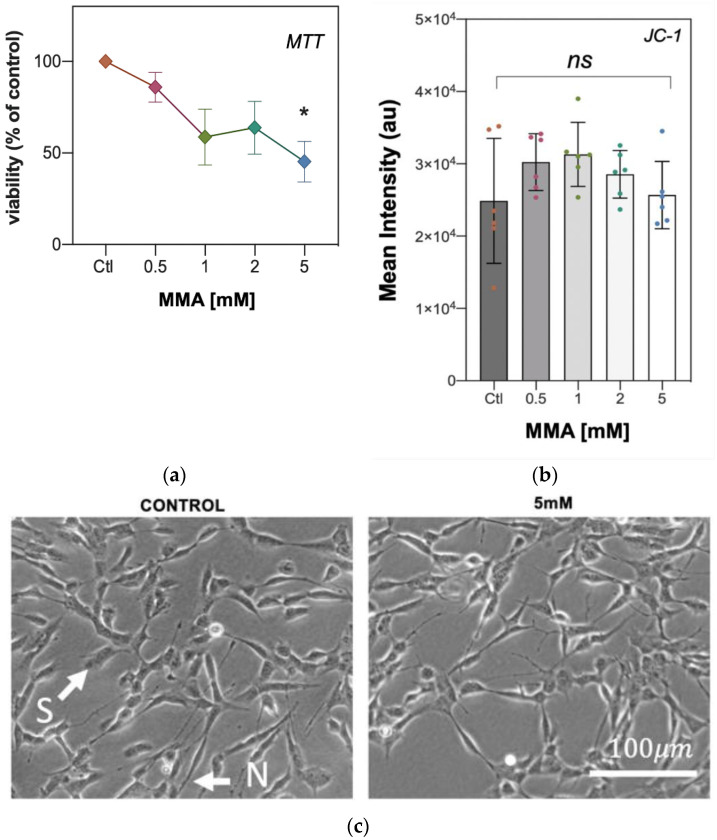
The effect on MMA treatment of SH-SY5Y cell viability as determined by the MTT asay. (**a**) MTT absorbance plotted as % of control. Error bars represent the mean ± SD, *n* = 3; (**b**) Phase-contrast micrograph of SH-SY5Y morphology following treatment with 5 mM MMA, S and N phenotypes are retained; (**c**) ΔΨm determination of SH-SY5Y cells as determined by JC-1 flourescence probe following treatment with MMA (0.5, 1, 2 and 5 mM). Error bars represent the mean ± SEM, *n* = 6. Statistical analysis was carried out using the Kruskal–Wallis test with Dunn’s multiple comparisons. * *p* = 0.0099.

The effect of the MMA-induced CoQ_10_ deficiency and loss of MRC complex II–III activity was assessed on SH-SY5Y neuronal cell viability by use of the MTT (3-(4,5-dimethylthiazol-2-yl)-2,5-diphenyl tetrazolium bromide) assay. Following treatment with MMA a progressive in the reduction in MTT reaching significance (* *p* = 0.010, Figure 2a) which induced a 54.7% loss in cell viability compared to control levels. Using phase-contrast microscopy, no significant changes in neurite length, cellular phenotype (S/N) or cell rounding were observed between MMA-treated and control cells (Figure 2b), indicating that the loss of viability post treatment with MMA may have resulted from as yet unknown causes. No significant difference was found between the protein concentrations of the control and 5 mM MMA-treated cell samples, 4.07 ± 0.22 mg/mL and 4.22 ± 0.40, respectively (mean ± SEM, *n* = 5).

The effect of the MMA-induced CoQ_10_ deficiency and loss of MRC complex II–III activity was also assessed on SH-SY5Y neuronal cell mitochondrial membrane potential (ΔΨm) via flow cytometry using the Ψm-specific probe, JC-1. However, MMA treatment was found to have no significant effect on neuronal cell ΔΨm (ns, *p* = 0.284, Figure 2b) compared to the control.

## 3. Discussion

To our knowledge, this is the first study to report the ability of MMA to induce a deficit in cellular CoQ_10_ status. This indicates the possibility that elevated levels of MMA, previously reported in methylmalonic acidemia [2] may have the potential to induce an endogenous CoQ_10_ deficiency in patients. In view of the severe neurological symptoms associated with methylmalonic acidemia [4] and the fact that neurons may be exposed to CSF MMA levels as high as 2.5–5 mM during crises [19,26], we used this study to investigate whether MMA at these concentrations may have the potential to induce a neuronal cell CoQ_10_ deficiency. The 75% decrease in cellular CoQ_10_ status following exposure of the neuronal cells to 0.5 mM MMA indicates the susceptibility of these cells to a MMA-induced CoQ_10_ deficiency and suggests the possibility that a deficit in CoQ_10_ status may be a contributory factor to the neurological dysfunction associated with this disorder. However, further studies will be required before this can be confirmed or refuted. In view of the dependence of MRC complex II–III activity upon endogenous CoQ_10_ status [20], the effect of the MMA-induced CoQ_10_ deficiency was assessed on the activity of this enzyme system. However, although the activity of complex II–III was decreased after neuronal exposure to all concentrations of MMA, a significant loss of activity was only observed following treatment with 2 mM MMA. The reasons for this are uncertain in view of the comparable levels of residual CoQ_10_ present in the neuronal cells following treatment with the different concentrations of MMA. However, the possibility arises that in addition to a deficit in CoQ_10_ status, the loss of complex II–III activity may also be the result of MMA-induced inhibition of either complex II or III activity, which may only be induced at higher concentrations of the acid. However, a study by Brusque et al. (2002) [19] also reported the ability of MMA to inhibit the activity of MRC complex II–III activity in rat cerebral cortex homogenates in the absence of a deficit in either individual complex II or III activity. Unfortunately, no assessment of CoQ_10_ was undertaken in the study by Brusque et al. (2002) [19]. However, the ability of MMA to act as competitive inhibitor of MRC complex II activity was reported. However, complex II activity was only inhibited by MMA (1, 2.5 and 5 mM) when 1 mM of succinate was used as the substrate concentration for the assay [19]. A concentration of 20 mM was used to assess MRC complex II–III activity in the present study and therefore, it is unlikely that the loss of enzyme activity may be attributable to MMA-induced complex II inhibition, although this has yet to be confirmed or refuted and the possibility arises that the loss of complex II–III activity may be attributable to other factors associated with MMA treatment such as OS or direct enzyme inhibition which may only occur at the higher concentrations of the acid [10,11,12,14].

Interestingly, although MMA was found to induce a 75% decrease in neuronal cell CoQ_10_ status together with a 61% decrease in MRC complex II–III activity (2 mM MMA), there appeared to be no apparent loss of ΔΨm as assessed by the JC-1 cationic fluorescent dye assay. A possible explanation for this result is that CoQ_10_ and complex II–III may have to be inhibited beyond 75% and 61%, respectively of control levels before the ΔΨm becomes impaired. Indeed, it has been reported that the activity of MRC complex III, a component of complex II–III has to be inhibited by approximately 80% of its original activity before oxidative phosphorylation becomes compromised in synaptic mitochondria isolated from rat brain [29].

Two previous studies have reported evidence of an association between methylmalonic acidemia and a deficit in CoQ_10_ status, one in patient fibrobasts [24] and the other in a mouse model of the disease [25]. However, in these studies the factors responsible for inducing the loss of CoQ_10_ status were not investigated, and the present study is the first to indicate that MMA may be capable of causing a diminution in the status of this isoprenoid. However, the mechanism by which this occurs is at present uncertain. The comparable levels of CS activity, the mitochondrial marker enzyme [27] between the control and MMA-treated cells suggests that the decrease in CoQ_10_ status does not reflect a loss in mitochondrial enrichment of the neurons. The possibility arises that MMA may be able to directly inhibit one or more of the enzymes involved in CoQ_10_ biosynthesis. The reported ability of MMA to inhibit MRC complex I activity together with complex II–III [19], the latter being confirmed in the present study, may also be an explanation for the decrease in CoQ_10_ status of the neuronal cells. The loss of MRC complex I and linked II–III activity following MMA treatment would be expected to reduce electron flow in the MRC and therefore, the requirement for the electron carrier, CoQ_10_ whose synthesis may then be suppressed as suggested in the study by Yudero et al. (2016) resulting in a decrease in the cellular level of this isoprenoid [30]. Furthermore, in view of the reports of OS in methylmalonic acidemia [31] together with its association with MMA treatment [10,11,12], the decrease in neuronal CoQ_10_ status observed in the present study may also result from either increased oxidative catabolism of the molecule or reactive oxygen species induced inhibition of the enzymes involved in CoQ_10_ biosynthesis [32]. The possibility also arises that MMA may sequester CoaSH resulting in a decrease in cellular acetyl-CoA availability which is required by the mevalonate pathway for the synthesis of CoQ_10_ [21]. A decrease in cellular acetyl-CoA availability has been previously suggested by Yubero et al., 2014 [33] as one of the possible mechanisms to account for an association between a CoQ_10_ and a glucose transporter deficiency. Further studies will be required to assess evidence for this putative mechanism of CoQ_10_ deficiency in methylmalonic acidemia.

Commensurate with the deficit in CoQ_10_ status and MRC complex II–III activity, a significant decrease in the reduction in MTT (*p* = 0.010) by the SH-SY5Y cells following exposure to 5 mM MMA was observed. Since the MTT assay assesses cellular metabolic activity as an indicator of cell viability, proliferation and cytotoxicity [34] from the results of the present study we cannot be certain as yet to the causes of the loss of reduction in MTT and further studies will be required to confirm or refute whether this reflects a loss in cell viability or cellular proliferation. However, the similar levels of CS activity, the mitochondrial marker enzyme [35] and cell protein following MMA exposure may indicate that differences in cell proliferation may not be solely responsible for the results of the MTT assay. Although the deficit in CoQ_10_ status and complex II–III activity appear to have contributed to the loss in cell viability, other factors should also be considered that may explain why a significant loss in cell viability was only observed following exposure to 5 mM MMA. However, at present, these factors have yet to be elucidated. Further, it should be noted that following addition of 5 mM MMA to the cell culture media there was a decrease in pH from 7.49 to 7.02, and although this did not appear to have a direct effect on the enzyme and MTT assays used in the present study it is at present uncertain whether this had a detrimental effect on MRC complex II–III activity and/or cell viability and this requires further investigation. The reported inhibitory effect of MMA on MRC activity should also be a consideration [19]. However, the maintenance of the ΔΨm following exposure to MMA suggests that impaired oxidative phosphorylation may not have been a contributory factor. Importantly, MMA treatment in animal and cell studies has been reported to induce an increase in lipid and protein peroxidation as well as to decrease the activity of the antioxidant enzyme, superoxide dismutase and the level of cellular antioxidant, glutathione [10,11,12,14,31]. Therefore, a MMA-induced increase in OS may have also contributed to the loss of neuronal cell viability, although this was not investigated in the present study. The MMA-induced decrease in cellular CoQ_10_ status would also be expected to have contributed to cellular OS in view of the important antioxidant function of this molecule [21].

It is important to stress that this study was conducted in SH-SY5Y human neuroblastoma cells, which, although possess many of the functional and biochemical properties of early sympathetic neurons, are heavily dependent upon glycolysis for ATP generation in contrast to primary neurons which rely on oxidative metabolism for energy generation [36,37]. Therefore, although SH-SY5Y cells are commonly used as a human neuronal cell model [36], further studies will be required before it can be confirmed or refuted whether MMA has the potential to induce a CoQ_10_ deficiency in methylmalonic academia patients. However, taking this caveat into the consideration, this study highlights the vulverability of neuronal cells to MMA-induced CoQ_10_ deficiency, with 0.5 mM MMA being suffient to induce an approximate 75% decrease in the level of this isoprenoid. This is especially pertinent since it has been reported that the level of MMA may be as high as 2.5–5 mM in the blood and cerebrospinal fluid (CSF) of methylmalonic acidemia patients during crisis, possibly being even higher in neuronal cells [26].

Currently, there is a paucity of information in the literature on the use of CoQ_10_ in the treatment of methylmalonic acidemia. However, CoQ_10_ treatment in conjunction with vitamin E has been reported to improve visual acuity in a 15-year-old patient with methylmalonic acidemia and optic neuropathy [38]. Furthermore, a significant improvement in glomerular filtration rate was reported in a mouse model of the condition following co-treatment of vitamin E and CoQ_10_ [25].

**Figure 3:**
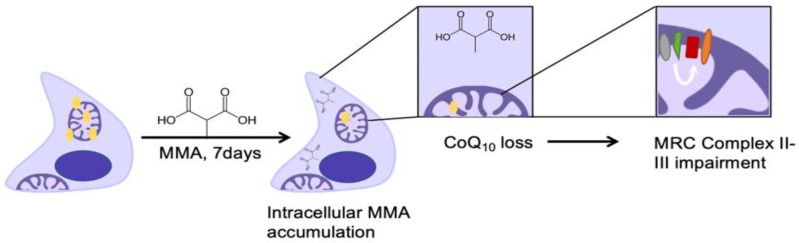
Schematic representation to show the effect of MMA exposure on SH-SY5Y neuronal cell CoQ_10_ staus and MRC complex II–III activity.

In conclusion, the results of this study have indicated the susceptibility of SH-SY5Y human neuroblastoma cells to MMA-induced CoQ_10_ deficiency which is also associated with a loss in MRC complex II–III activity (Figure 3). Although this study was undertaken in SH-SYSY human neuroblastoma cells, a widely used model for human neuronal cells, it remains to be determined whether the findings of this study are translatable to neurons in vivo. Therefore, the possibility arises that the cellular CoQ_10_ status of methylmalonic acidemia patients exposed to concentrations of MMA ≥ 0.5 mM may be decreased compared to unaffected individuals, although this needs to be confirmed or refuted. The assessment of the endogenous CoQ_10_ status of these patients may prove informative. However, further studies are required to determine whether this potential deficit in neuronal CoQ_10_ status contributes to the disease pathophysiology of methylmalonic acidemia.

## 4. Materials and Methods

### 4.1. Cell Culture

The SH-SY5Y neuroblastoma cell line was purchased from the European Collection of Cell Cultures (Health Protection Agency, Salisbury, UK). This is thrice cloned cell line with a neuroblast-like morphology. The SK-N-SH cell line, from which these cells were originally cloned, has demonstrated various neurotransmitter activities: dopaminergic, acetylcholinergic and adenosinergic [39].

SH-SY5Y cells (human bone marrow neuroblastoma; ATCC CRL-2266) with epithelial and neuroblast-like phenotypes were cultured in Dulbecco’s Eagles Modified Medium (DMEM) (Sigma Aldrich), supplemented with 10% Fetal Bovine Serum (FBS) and 1% Penicilin Streptomycin (P/S) at 37 °C. Cell densities modified based upon incubation periods.

The concentrations of MMA used in the present study are based on those reported in the blood and CSF of methylmalonic acidemia patients which have been reported to be ≥2.5 mmol/L during acute metabolic crisis, [19,26]. In view of the fact that the half life of cerebral ubiquinone species (coenzyme Q_9_ and CoQ_10_) have been reported to be 90 h (3.75 days), and to ensure that the majority of the endogenous CoQ_10_ synthesized prior to MMA treatment had been degraded, a time course of 7 days was selected for this study [40]. For these experiments stock solutions of MMA were prepared by dissolving MMA in Milli-Q water.

At 70–90% confluence, cells were harvested, washing twice with phosphate buffer saline (PBS) followed by the addition of trypsin (3 mL). Cells were seeded in plastic 96-well plates at a density of 20,000 cells/well or T75 flasks at a density of 150,000 cells/mL prior to treatments with MMA. MMA treatment was carried out in DMEM at concentrations of 0.5, 1, 2 and 5 mM and SH-SY5Y cells were treated with MMA for 7 days without a media change during the 7 day incubation period. As the MMA was dissolved in Milli-Q water, control cells were treated with the vehicle Milli-Q water (50 µL). The pH of the cell culture media was determined following addition of MMA to assess whether there was any change in the pH of the environment to which the cells were exposed during culture. Following the inclusion of MMA in the cell culture media the pH changed from 7.49 to 7.76,7.68,7.49 and 7.02 after the addition of 0.5, 1, 2 and 5 mM MMA, respectively.

### 4.2. High-Performance Liquid Chromatography (HPLC)

Total cellular CoQ_10_ status was quantified using a HPLC UV detection method as previously described by Duncan et al. 2005 [41]. CoQ_10_ was extracted from the cell samples according to the method of Boitier et al. (1998) [42] using a solvent of hexane: ethanol (5:2, *v*/*v*). Cells samples together with the internal standard (di-propoxy CoQ_10_) were vigorous mixed in eppendorf tubes with the solvent and subjected to centrifugation (5 min × 5000× *g* at 4 °C) and the upper hexane layer was retained for analysis. The lower aqueous layer was extracted two more times with solvent and the resulting upper hexane layers were combined and evaporated under nitrogen gas. During the extraction procedure all cellular ubiquinol should be oxidised to CoQ_10_. The sample was then re-suspensed in HPLC-grade ethanol before being injected onto the HPLC. separation was achieved using a reverse phase C18 column (150  ×  4.6, HPLC Technology, Welwyn Garden City, UK), using a mobile phase composed of methanol/ethanol/perchloric acid (700:300:1.2) at a flow rate of 0.7 mL/min. CoQ_10_ was detected at 275 nm using a PG-975-50 UV/VIS detector (Jasco, Essex, UK). Cellular CoQ_10_ content was estimated by comparison with a standard solution of CoQ_10_.The inter and intra assay coefficient of variation (CV) for this assay using the cell samples were 2.6% (331 nmol/L; *n* = 5) and 3.5% (371 nmol/L; *n* = 5), respectively. Upon injection of 2 and 5 mM MMA into the HPLC system, no peaks were detected on the HPLC chromatogram at 275 nm, indicating that this metabolite does not co-elute with or influence the accurate determination of CoQ_10_. All quantification in pmol/mg was expressed relative to total protein content via the Lowry Assay.

### 4.3. Spectrophotometric Enzyme Assays

Enzymatic determinations were undertaken at 30 °C using a Uvikon XL spectrophotometer (Northstar, Leeds, UK).

The specific activities of the MRC enzymes were determined according to previously described methods:

Complex II/III (succinate dehydrogenase: cytochrome *c* reductase; EC. 1.3.5.1 + 1.10.2.2) was measured by the antimycin A sensitive reduction in cytochrome *c* at 550 nm according to the method developed by King [43]. Antimycin A is a specific inhibitor of complex III activity, and therefore in the assay, the enzymatic rate pre antimycin A addition is subtracted from the activity post antimycin A addition to provide a complex II–III-specific enzyme activity [43].

CS activity was determined by the liberation of free CoA with DTNB (5,5′ dithio-bis (2-nitrobenzoic acid) which forms a stable conjugate that can measured at 412 nm according to the method of Shepherd and Garland [35]. Complex II–III activities were expressed as a ratio to CS activity to correct for the mitochondrial enrichment of the samples [44].

In the absence of SH-SY5Y cells 5 mM MMA was not found to affect the complex II–III or CS enzyme assays.

### 4.4. MTT Assay

The MTT (3-(4,5-dimethylthiazol-2-yl)-2,5-diphenyl tetrazolium bromide) assay was used to assess to assesses the cellular metabolic activity (an indicator of cell viability, proliferation and cytotoxicity of the SH-SY5Y human neuroblastoma cells. This colormetric assay is based on the ability of the mitochondrial enzyme NADPH-dependant cellular oxidoreductase enzymes to reduce MTT to its purple coloured insoluble formazan which absorbs at 590 nm which is proportional to the NADPH-dependent reductase cellular activity, and accordingly, proportional to the number of proliferating and viable cells [34]. Post addition of trypan blue solution, dye excluding cells were counted using a haemocytometer. Cells were then plated and treated with MMA as previously described. MTT solution (20 µL) was added to each well of the 96 well plate post a 7 day incubation with the MMA. After a 3–4 h incubation with MTT at 37 °C, the formazan product was solubilized using 100 µL of DMSO and the absorbance of each well was read at 570 nm on a CLARIOstar^®^ Plus.

In the absence of SH-SY5Y cells, 5 mM MMA was not found to affect the MTT assay.

### 4.5. Flow Cytometry

JC-1 (5,5,6,6′-tetrachloro-1,1′,3,3′-tetraethylbenzimi-dazoylcarbocyanine iodide) is a cationic fluorescent dye that accumulates in energized mitochondria. JC-1 assay measures the mitochondrial membrane potential, in that the fluorescent intensity of the dye indicates an increase or decrease in mitochondria membrane potential. After 7 day incubation with MMA, neuroblastoma cells were washed with 20 µL of PBS and stained with JC-1 (1:2000 dilution), cells were gently mixed and left to incubate at 37 °C, 5% CO_2_ for 30 min. After incubation, cells were washed with PBS (20 µL), and trypsinized before being analysed on a flow cytometer using 488 nm excitation [45].

### 4.6. Protein Determination

Protein concentrations of the SH-SY5Y cell samples were determined by the method of Lowry and colleagues (1951) [46] using BSA as a standard.

### 4.7. Determination of Intracellular MMA Concentrations

The cellular levels of MMA were determined in SH-SY5Y cells by stable-isotope dilution and liquid chromatography–tandem mass spectrometry according to the method of Blom et al. (2007) [47]. The cell samples were frozen and thawed 4 times in liquid nitrogen followed by vigorous vortex mixing. The samples were then diluted 1 in 200 with Milli-Q water. The diluted samples were mixed with a solution containing a stable isotopically labelled MMA standard and then subjected to ultrafiltration. After removal of water, hydrochloric acid (HCL, 1 mM) and butanol were added to the sample to esterify the residue. Solvent and HCl were removed and the residue was re-dissolved in mobile phase prior to analysis by liquid chromatography–tandem mass spectrometry. MMA was quantified according to known standards.

### 4.8. Statistical Analysis

Statistical analysis was performed using GraphPad Prism (Version 8.4.1. 460 for macOS). A *p* value of < 0.05 was considered to be statistically significant. Before performing analyses, distributions of data were checked for normality with the Shapiro–Wilk test. Measurement data were expressed as the mean ± standard error of the mean (SEM). For all data deemed non-parametric, the Kruskal–Wallis test with Dunn’s multiple comparison was used to evaluate statistical significance between control and MMA-treated cells. In all cases, *p* < 0.05 was considered significant.

## Abbreviations

MMA	Methylmalonic acid
mut^0^	Complete defect of MCM activity: OMIM 251000
mut^-^	Partial defect of MCM activity, OMIM 251000
BBB	Blood–brain barrier
2-MCA	2-Methylcitrate
HPLC	High-performance liquid chromatography
IMM	Inner mitochondrial membrane
